# Effect of disaster training on knowledge regarding flood risk management amongst families with older people

**DOI:** 10.4102/jamba.v14i1.1262

**Published:** 2022-08-18

**Authors:** Fatmah Fatmah

**Affiliations:** 1 Disaster Management Program, School of Environmental Science, Universitas Indonesia, Jakarta, Indonesia

**Keywords:** disaster preparedness, flood, landslides, knowledge, older people, training

## Abstract

The elderly population is of utmost importance amongst vulnerable populations during disasters because they experience reduced functional abilities, cognitive disturbance, dementia, weak physical conditions and various degenerative diseases. This study aimed to assess the effect of disaster preparedness training on knowledge regarding flood disaster preparedness and management in families with older people. This study was conducted using a quasi-experimental pre-post design with 30 participants in natural hazard preparedness training using purposive sampling. The results of this study showed a significant change in general knowledge on disaster and flood (12.9 and 20 points, respectively). Disaster preparedness practice was good, as reflected in actions performed before, during and after disaster. Before a flood occurs, families prepare a disaster preparedness bag for important documents as well as logistics (e.g. food) for emergencies and equipment for evacuation whilst also storing valuable goods in a safe place. During floods, families prioritise older people’s evacuation whilst seeking information about the flood through neighbours, walkie-talkies, handphones, television and radio as well as ensuring that the necessary logistics are taken care of. After the disaster, families clean their properties, provide clean water and toilet facilities for the family, check the health of family members that may be impacted by the flood and make sure that all electrical panels are safe. It is concluded that disaster training affects the knowledge of flood management in families with older people.

## Introduction

Indonesia is an archipelagic country that is located within the Pacific Ring of Fire, making it prone to disasters (Kontan 2021). Therefore, the country is at risk of various types of disasters including volcanic eruptions, earthquakes, floods, landslides and storms. The occurrence of a disaster cannot be predicted with certainty, so people must always be prepared to deal with disasters. A total of 1829 natural hazards have occurred in Indonesia from early 2021 to the beginning of September 2021, with floods (750 cases) and landslides (346 cases) as the main disasters (Kontan 2021). *Law No. 24 of 2007* on disaster management has declared that older people are one of the groups most vulnerable to disasters (National Disaster Management Agency 2007). Most disaster victims consist of children and other vulnerable groups, such as women (Tearne et al. 2021) and older people (Guddo 2020). Older people may face moderate to high hazard levels during a disaster because of their limited physical capacity, reduced sensorial capacity and presence of degenerative diseases (Cornell, Cusack & Arbon 2012; Yodsuban & Khanitta 2021). Therefore, it is important to improve the knowledge and behaviour of older people and their families who live in hydrometeorological disaster-prone areas to become better prepared to deal with a disaster.

It is thus critical to understand the concepts of disaster preparedness and management. According to the National Disaster Management Agency (2017), disaster preparedness refers to the activities performed before disasters so as to develop operational capacity and facilitate effective responses when a disaster strikes. On the other hand, disaster management comprises efforts or activities carried out in the context of disaster prevention, mitigation, preparedness, emergency response and recovery, which are performed before, during and after a disaster (Kyoo-Man 2020; The Indonesian Minister for Public Works and Human Settlements 2017; United Nations Office for Disaster Risk Management [UNDRR] 2022; United Nations Office for Outer Space Affairs [UNOOSA] 2022). Meanwhile, disaster mitigation is a series of efforts to reduce disaster risks through physical development, increased awareness and development of the ability to deal with disaster threats. The objectives of disaster mitigation may be achieved through education, counselling and training. Mitigation and preparedness should be established before a disaster occurs, that is, during the predisaster phase (Raikes et al. 2019). Training on disaster preparedness as a form of disaster mitigation and as an effort to prepare for disasters is needed to increase public knowledge and awareness of disaster risks in the area where they live (Centers for Disease Control and Prevention [CDC] 2014; Kawasaki et al. 2022; National Disaster Management Agency 2016). People who are well trained in disaster preparedness and mitigation are prepared to have good public awareness to reduce disaster risks and to collaborate well with agencies responsible for disaster management and other external parties before, during and after a disaster (Sogand et al. 2019). Disaster preparedness refers to the activities performed before disasters to develop operational capacity and facilitate effective responses when a disaster strikes (Ali & Ismaila 2020; The Indonesian National Disaster Management Agency 2017). Older people with good attitudes are those who are ready to deal with disasters based on good knowledge of the signs of disaster, making them always ready to evacuate at any time. Disaster preparedness has the aim of ensuring appropriate and rapid efforts in dealing with disaster events. Knowledge is the key factor that can influence the attitude and awareness of the community to be ready and prepared in dealing with disasters. One of the stages of disaster preparedness is education and training.

The West Java province, as the most populous island in the country, is prone to earthquakes, floods, landslides and storms, whilst the city of Depok, one of the cities of West Java, has declared a disaster alert status for floods, landslides and strong winds for a period of 14 days (01 January to 14 January 2020) as a response to increased disaster events. Depok had experienced twelve floods and six landslides with no fatalities from 2007 to 2015 (The Indonesian Ministry of Agraria and Spatial Planning 2006). There was an increased incidence of floods in the subdistricts of Pancoran Mas and Mampang (Depok City) in the last three years as a result of poor drainage systems and high precipitation (Depok City Fire and Disaster Management Service 2020). These areas have a high potential for flooding caused by a high intensity of rain, which leads to the overflow of river water triggered by inadequate slope inclination and river flow capacity. This is worsened by the fact that the two areas do not have a good drainage system, leading to a potential yearly flooding event.

Based on the data in the flood emergency contingency of Depok City Fire and Disaster Management Agency, several flood incidences had been recorded in the subdistrict of Pancoran Mas, specifically, a 1.5 m flood in the Marine Housing Complex, RW 6 Rangkapan Jaya Baru urban village (250 people affected), a flood caused by the overflow of the stream Krukut in the Arsip Nasional Housing Complex of the Mampang urban village and a landslide in the Rangkapan Jaya urban village (Dadan 2021; Depok City Fire and Disaster Management Service 2020; Depok District of Public Works and Human Settlements 2020). Based on high risk of flood in the study area and the older people exposed to it, a training session on flood disaster preparedness for families with older people was conducted on 30 August 2021 with 30 *posbindu* [integrated service post for older people] subdistrict and village cadres, as well as older people working groups to improve their preparedness to deal with disasters, especially concerning those with increased vulnerability. The purpose of the training was among increase the knowledge of disaster preparedness among older people and their families who live in flood-prone areas.

## Materials and methods

This study used a quasi-experimental design without a control group and a purposive sampling technique (Sastroasmoro & Ismael 2014).

The research question of the study was how much the respondents’ knowledge changed after being given disaster management training. To answer the research question, a one-day training session on flood preparedness for families with older people was conducted on 30 August 2021 at the Pancoran Mas Subdistrict Office. Disaster preparedness education was carried out using printed media (leaflets and flipcharts) and visual media (video) developed by the research team.

The pre-post test questionnaire was developed using several standard questionnaires whose validity has been tested. The questionnaire consisted of 50 questions, with four on sociodemographic characteristics, nine on general knowledge about natural hazards, seven on flood knowledge and 20 on practices of older people before, during and after a flood. The question items listed in the pre-post test were as follows: sociodemographic characteristics (marital status, age, education background, primary job, length of time as a cadre and whether or not they had attended training before), general knowledge of natural hazards (definition, types, examples of disasters, impacts, natural hazard-prone groups, preparedness of families and of the older people), specific knowledge of flood (definition, causes, impacts, flood prevention, mitigation efforts and actions taken before, during and after flood) and practices regarding preparedness of the older people before, during and after flood (Fatmah 2012, 2013, 2020).

The mean score for knowledge was measured twice: before and after the test. The purpose of the pre- and post-tests was to assess the changes in knowledge of families with older people in flood preparedness. The training was planned in advance through coordination with the Pancoran Mas district, Depok City. They recruited all the selected participants from six villages who met the criterion. There were 30 trainees, consisting of 27 women and three men, who had a role as older people working-group members of the subdistrict, older people working-group members of urban villages and family with older people. They came from six villages from the subdistrict of Pancoran Mas, Depok City, West Java province and Indonesia (Depok Jaya, Depok, Pancoran Mas, Mampang, Rangkap Jaya and Rangkap Jaya Baru).

The data were analysed for frequency distribution, minimum and maximum values and study variable means. Scores of preparedness knowledge and practice before and after training were analysed using an independent *t*-test. A *p* < 0.05 was considered significant. Data analysis was performed using the SPSS version 21 for Windows.

### Ethical considerations

Ethical clearance for this study was obtained from the Health Research Ethics Commission of the National Institute of Health Research and Development, Ministry of Health of the Republic of Indonesia (reference number: LB.02.01/2/KE.374/2021). An informed consent form was signed by the respondents in the third week of August 2021.

## Results

### Sociodemographic characteristics of respondents

Table 1 presents the sociodemographic characteristics of respondents. Most respondents were *posbindu* cadres (83.3%), and the remaining were members of the subdistrict and urban village older people working-groups. Most of the training subjects were women (27 out of 30 participants). Most of the respondents were not yet older people, with the oldest being 73 years old and the youngest being 30 years old (on average 51.9 years old). Over three-quarters of the respondents were married, and most had obtained a high school degree or equivalent. Almost all respondents were no longer working, but a small proportion still worked as traders and entrepreneurs. Over half of the total respondents had previous nutrition and health-related trainings, including trainings with older people, particularly on coronavirus disease 2019 (COVID-19), stunting, family nutrition awareness, food and nutrition, clean and healthy lifestyles, nutrition for children under five years of age, hypertension, Alzheimer’s disease, diabetes, *posbindu* cadre training, solid food introduction to infants, older people exercise, cervical cancer and health care.

**TABLE 1:** Sociodemographic characteristics.

Variable	*n*	%	Mean	± SD	Min – Max
**Marital status**					
Married	24	80	-	-	-
Widow or widower	6	20			-
**Gender**					
Female	27	90	-	-	-
Male	3	10	-	-	-
**Age (years old)**			51.9	± 9.8	30–73
< 60	23	76.7	-	-	-
≥ 60	7	23.3	-	-	-
**Final education level**					
Low: (≤ 9 years)	5	16.7	-	-	-
Medium: (> 9 years)	25	83.3	-	-	-
**Working status**					
Work	3	10.0	-	-	-
Entrepreneur	2	6.7	-	-	-
Trader	1	3.3	-	-	-
Does not work	27	90.0	-	-	-
**Ever attended training before**					
Yes	20	66.7	-	-	-
No	10	33.3	-	-	-

### Changes in knowledge of disasters and floods

The mean score of respondents’ knowledge of general and flood disasters increased after the training (see Table 2). The knowledge about floods exhibited the highest increase, followed by knowledge of general disasters. The three types of knowledge presented a significant difference before and after the training intervention. The mean changes in knowledge of disasters and floods by sociodemographic characteristics are shown in Table 3. There was no correlation between increased knowledge of general and flood disasters with marital status, gender, age, education background, employment status and participation in the previous health and non-health-related trainings before (*p* < 0.01). However, respondents who had attended training before tended to have a slightly higher score on the general disaster knowledge than those who had never participated in such training before, except knowledge on floods (Table 3).

**TABLE 2:** Changes in knowledge about disasters in general and floods.

Variable	Score						*p*
	Pre-test		Post-test		Difference		
	Mean	± SD	Mean	± SD	Mean	± SD	
Knowledge on general disaster	69.6	± 19.9	82.5	± 11.0	12.9	± 18.2	0.001
Knowledge on floods	52.4	± 18.9	72.4	± 15.8	20.0	± 14.1	0.001

**TABLE 3:** Mean change in knowledge of general disasters and floods, based on sociodemographic characteristics.

Variable	*n*	Knowledge on general disasters			Knowledge on flood		
		Mean	± SD	*p*	Mean	± SD	*p*
**Marital status**							
Married	24	13.4	± 19.9	0.791	21.1	± 13.3	0.412
Widow or widower	6	11.1	± 9.4		15.7	± 17.7	
**Gender**							
Male	3	2.8	± 4.8	0.316	11.8	± 15.6	0.295
Female	27	14.0	± 18.8		20.9	± 14.0	
**Age (years old)**							
< 60	23	15.0	± 19.8	0.253	22.5	± 13.5	0.078
≥ 60	7	6.0	± 9.3		11.8	± 14.0	
**Education level**							
Low (< 9 years)	5	22.5	± 14.9	0.201	23.5	± 23.5	0.550
Medium (≥ 9 years)	25	11.0	± 18.4		19.3	± 12.1	
**Working status**							
Work	7	11.9	± 7.8	0.870	23.5	± 16.3	0.460
Does not work	23	13.2	± 20.4		18.9	± 13.6	
**Had ever participated in the previous training**							
Yes	20	14.2	± 20.1	0.603	18.2	± 14.8	0.342
No	10	10.4	± 14.1		23.5	± 12.7	

The proportion of correct answers on general knowledge of disasters and flood before and after the training is shown in Table 4. The largest increase in the proportion of correct answers on general knowledge of disasters was observed for the types of disasters (40%), followed by impact of disasters and examples of disasters. The questions that were most frequently answered correctly in the pre-test were the ones on older people, including whether older people are included in the vulnerable groups during a disaster and if older people need to prepare themselves for disasters. The item that had the highest proportion of correct answers on knowledge of floods concerned the actions that should be taken by a family when a flood occurs. The item with the lowest proportion of correct answers was the question on flood prevention and mitigation efforts. In general, the percentage of correct answers in the flood knowledge section was lower than 40%.

**TABLE 4:** Comparison of the proportion of correct answers regarding knowledge of general natural hazards and floods, before and after training.

Knowledge on general natural hazards and floods	Proportion of correct answers (%)		Difference
	Pre	Post	
**Knowledge on general natural hazards**			
Able to mention three definitions of disaster	30.0	70.0	40.0
Able to mention three types of disaster	36.7	50.0	13.3
Able to mention at least three examples of natural hazards	56.7	76.7	20,0
Able to mention three types of disaster impact	66.7	96.7	30.0
Older people are vulnerable to disaster	93.3	100.0	6.7
Older people, children, pregnant mothers and lactating mothers are groups vulnerable to natural hazards	76.7	86.7	10.0
Older people need to prepare for natural hazards	93.3	100.0	6.7
Preparedness of older people and their families for natural hazards	63.3	73.3	10.0
**Flood**			
Able to mention four definitions of disaster	20.0	33.3	13.3
Able to mention three causal factors of floods	60.0	80.0	20.0
Able to mention three impacts of floods	66.7	90.0	23.3
Able to mention four actions before the flood	13.3	46.7	33.4
Family actions taken during flood	40.0	93.3	53.3
Family actions taken at post-flood	60.0	93.3	33.3
Able to mention four flood prevention and control efforts	26.7	30.0	3.3

### Practices of families with older people in dealing with flood

The first thing the respondents did before and when facing a sudden flood was to move electronic goods and household appliances to a higher place, evacuating themselves and family members to a higher floor of the house and evacuating to the house of a relative or someone else with a house located outside of the flood area. The first action taken by the respondent’s family when the flood suddenly entered the house was to evacuate the older people to a safer place. When asked whether the respondent felt anxious or worried during the previous flood, over half of the respondents stated that they felt anxious because there were older people and toddlers in the house, worried that the house would be completely submerged and panicked. Actions taken by respondents before the flood occurred included preparing a disaster preparedness bag containing important or valuable documents, logistics items (e.g. food) for emergencies, preparing equipment for evacuation and planning or placing valuables in a relatively safe place. The respondents also sought information related to the flood from neighbours, through walkie-talkies, cell phones, televisions and radios. They also ensured the availability of enough food and logistics supplies, ensured that all their family members were safe and evacuated to a safer place than that where the flood struck.

Almost all respondents stated that they prioritised older people during flood because of older people’s physical limitations, which prevent older people from performing quick actions to save themselves from the flood. When the flood had reduced, most respondents cleaned their houses from the mud, dirt and waste carried by the flood, provided clean water and toilet facilities for family members, checked the health of the family members that may have been affected by the flood and ensured that all electrical panels in the house were safe for use after the flood. Usually, families evacuated to the second floor of their own house, went to a neighbour’s house or relative’s house located outside the flooding area or went to a public building that was safe from the flood.

The emergency response plan prepared by the respondent in the event of a flood was to prepare first aid medicine at home, prepare equipment for self-rescue or emergencies and have large enough food stocks to survive during the flood. Most respondents received early flood warnings from the neighbourhood, which facilitated receiving the latest flood information. The media used for sharing information were WhatsApp groups, announcements from the mosque or Islamic prayer room speakers, Instagram, Facebook and other social media. Few respondents had attended training on natural hazard preparedness at the subdistrict level in 2019 and 2020. Most respondents expected that the people in their respective neighbourhoods had cleaned the waste from rivers or streams. The Depok City Government regularly normalises or dredges shallow rivers, repairs roadside waterways, provides flood education to the community and enhances infrastructure to anticipate future disasters.

## Discussion

Older people are a vulnerable group during disasters, in addition to adolescent girls, pregnant women, breastfeeding women, children and people with disabilities (Gill, Ekbal & Bruce 2020; Hildayanto 2020; Kar 2016). Older people are classified as vulnerable to natural hazards because of their reduced physical abilities and health status, reduced mobility, decreased sensory awareness and increased risk of morbidity before, during and after disaster situations. Specific protection measures need to be implemented during a disaster to enable the vulnerable groups to survive disaster and postdisaster situations. One of the specific efforts to reduce disaster risk is to increase preparedness and mitigation when dealing with disasters. Preparedness comprises organised, real and effective steps to anticipate disasters and is included in the predisaster cycle (Lesley 2017).

Increasing public knowledge and awareness on disasters is necessary for a good preparedness level in the community. This knowledge and awareness will influence how the people deal with disaster situations before, during and after a disaster regarding themselves, their families, older people and other vulnerable groups. The ability of the family to assist older people in a disaster situation is of utmost importance. Several studies have shown a significant relationship between family support and preparedness of elderly people in dealing with natural hazards (Dodon 2013; Erita, Donny & Adventus 2019). Family preparedness in assisting older people in dealing with disasters is a form of family care (Nurhidayati & Bahar 2018). The flood preparedness practice of older people was assessed at the beginning of the study. The objective was to obtain an overview of the practice of older people before, during and after the flood. This study focused on flood disasters because floods occupy the highest order of natural hazards in the city of Depok. There were several flood-prone areas in Depok City from 2018 to 2020. Depok’s flood vulnerability index ranged from low (46.4%) to moderate (36.5%) (The Indonesian Ministry of Agraria and Spatial Planning 2006). In 2021, Depok City had a moderate level of disaster risk (82.56 score) (National Disaster Management Agency 2021).

In general, the preparedness practice of families with older people dealing with flood is adequate from the perspective of the disaster cycle (before, during and after a disaster). Families with older people have taken appropriate action to evacuate the older people to a safer place. Information support is also provided by the family to older people by explaining actions that need to be taken before the flood. The family also prepares equipment that may be needed if a flood strikes, such as a disaster preparedness bag and food supplies. The disaster preparedness bag prepared by families with older people has to contain medications, first aid kits, clothing, blankets, emergency lamps or torches, batteries and so on (Erita, Donny & Adventus 2019). The findings of this study are in line with a study on the preparedness of families with older people in facing potential volcanic eruptions in Klaten (Lenawida 2011; Nurhidayati & Bahar 2018). In addition, this study concurs that information needs to be given repeatedly to older people considering potential age-related changes in their cognitive function (Fatmah 2010).

Efforts to improve older adults’ preparedness for floods should be done by involving them or their family members in regular training and seminars and by updating knowledge and information which can be directly accessed by them. As also found in this study, the increased knowledge of families with older people will become an important part of the efforts to increase disaster preparedness (Jannah, Daniah & Nur 2021). Disaster warning systems used by families with older people include WhatsApp groups at the neighbourhood level. Announcements from mosque or Islamic prayer room speakers and information from Internet sources such as Instagram, Facebook and other social media can be used to warn people, which could lead to faster evacuation. This is also emphasised in the findings of a study on flood preparedness in the city of Bekasi (Akhirianto 2018).

General knowledge of disasters and flood was significantly related to the results obtained before and after the intervention. No significant relationship was found between the changes in the two types of knowledge and the sociodemographic characteristics. This may be a result of the homogeneity of respondents regarding their educational background, gender and age group, where most respondents had graduated from the secondary education level, were female and were in the pre-older people group. This finding is supported by several studies that show a relationship between age, education, gender and knowledge (Islam, Chakrabarti & Dirani 2014; Nastiti, Rafiah & Acim 2021; Oktarina, Fachrudi & Made 2009). The higher the level of maturity, the higher the ability to deal with issues. A higher education also facilitates better information absorption, which in turn will impact the level of knowledge acquisition. Women consider disaster events or threats as a more serious and riskier situation, and are more involved in disaster mitigation and preparedness activities than men, especially in home-centred activities (World Health Organization 2002). Knowledge is gained after people use their senses (sight, hearing, smell, taste and touch) to identify a specific object (Notoatmojo 2007). Changes in the knowledge of disaster and floods as a result of the training are in the cognitive domain of knowledge and understanding. As also found by Anderson and Krathwohl (2017), our training has not yet reached the domain of application, analysis, synthesis and evaluation because it has not been made available to families and communities.

In general, the respondents’ knowledge of floods is good. This can be seen from the fairly high proportion of correct answers (score higher than 60) to several questions. The questions most frequently answered correctly were associated with older people being amongst the most vulnerable groups in a disaster, older people needing to be prepared for a disaster, three causes of a flood, three effects of a flood and family actions taken after a flood. This fairly good knowledge may be a result of the experience of dealing with floods that frequently strike the areas where the respondents live, leading to a better understanding of floods. In addition, all respondents are involved in older people working-groups of the urban village and subdistrict, as well as in the *posbindu*, where they are daily exposed to issues faced by older people. Good knowledge of floods also links to the practice of disaster preparedness in families with older people in the disaster management phases (before, during and after flood). The findings of this study support those of two previous studies showing that knowledge has positive effects on community preparedness practices for flood (Firmansyah, Rasni & Rondhianto 2014; Puspitasari, Lukita & Rismayanti 2021). Knowledge of flood preparedness can influence the attitude and attention of the community to the extent that they are always alert in anticipating potential disasters, especially people who live in areas prone to natural hazards (Teja 2021). Despite the valuable contribution of this study towards disaster preparedness, this study has many limitations. This study does not include a control group for comparison with the intervention group. In addition, the small sample size with a homogeneous educational background, age and gender may also influence the results. This calls for future studies on disaster preparedness to close the current identified limitations from this study.

## Conclusion

It is concluded that the knowledge and practice of cadres and members of older adult working-groups who have older adults as family members regarding flood and landslide disaster management are quite good, especially in the efforts to evacuate the older adult first during a natural hazard. It is further concluded that knowledge of floods and landslides increased significantly after the training. However, no significant relationship was recorded between the respondents’ sociodemographic characteristics and the increased knowledge of floods and landslides or whether respondents have attended any previous training. Nevertheless, there is a tendency of respondents who had previous training to have a higher score compared to those who had not received such training. It is thus necessary to hold flood preparedness training for families with older adults to increase flood disaster literacy, especially amongst older adults and other groups who are vulnerable to disasters in the community. The objective is to increase alertness in anticipating floods, which may occur at any time. This training also needs to be replicated in other areas prone to disasters by involving the community and disaster management authorities at the subdistrict, district or city and national levels.

## References

[CIT0001] Akhirianto, N.A., 2018, ‘Community knowledge and preparedness for flood disaster in Bekasi City (case study: Pondok Gede Permai Housing)’, *Jurnal Alami* 2(1), 63–72. 10.29122/alami.v2i1.2704

[CIT0002] Ali, M.A. & Ismaila, R.A., 2020, ‘Public perception and attitudes to disaster risks in a coastal metropolis of Saudi Arabia’, *International Journal of Disaster Reduction Risk* 44, 1–9. 10.1016/j.ijdrr.2019.101422 PMC7104059 32289014

[CIT0003] Anderson, L.W. & Krathwohl, D.R., 2017, *Foundation framework for learning, teaching, and assessment*, Pustaka Pelajar, Yogyakarta.

[CIT0004] Fatmah, F., 2010, *Geriatric nutrition*, Erlangga Publisher, Jakarta.

[CIT0005] Fatmah, F., 2012, ‘Increased knowledge and skills of cadres in measuring the height of the elderly, counseling on balanced nutrition and hypertension in Grogol Petamburan District, West Jakarta’, *Media Medika Indonesiana* 46(1), 53–60.

[CIT0006] Fatmah, F., 2013, ‘The effect of training on the improvement of technical knowledge and skills counseling on obesity and hypertension for *posbindu* cadres, Depok City’, *Makara Seri Kesehatan* 17(2), 49–54. 10.7454/msk.v17i2.3026

[CIT0007] Fatmah, F., 2020, ‘Training program to support *posbindu* cadre knowledge and community health centre staff in the Geriatric nutrition service’, *ASEAN Journal of Community Engagement* 4(2), 499–518. 10.7454/ajce.v4i2.1051

[CIT0008] Centers for Disease Control and Prevention (CDC), 2014, *Disaster preparedness and response: Complete course, Facilitator guide*, 1st edn., CDC, Atlanta, GA.

[CIT0009] Cornell, V.J., Cusack, L. & Arbon, P., 2012, ‘Older people and disaster preparedness: A literature review’, *The Australian Journal of Emergency Management* 27(3), 49–53.

[CIT0010] Dadan, R., 2021, *Presentation of proposals for handling urgent needs for FY 2021 in the water resources sector*, Depok City Public Works and Spatial Planning Service, Depok.

[CIT0011] Depok City Fire and Disaster Management Service, 2020, *Flood emergency contingency TWG map FY 2020*, Depok City Fire and Disaster Management Service, Depok.

[CIT0012] Depok District of Public Works and Human Settlements, 2020, *Data recapitulation on natural disaster 2020*, Depok District of Public Works and Human Settlements, Depok.

[CIT0013] Dodon, 2013, ‘Indicators and behavior of community preparedness in traditional settlements in anticipation of various phases of flood disaster’, *Jurnal Perencanaan Wilayah dan Kota* 24(2), 125–140.

[CIT0014] Erita, D.M. & Adventus, M.R.L.B., 2019, *Emergency and disaster management learning materials book*, The Indonesian Christian University, Jakarta, p. 6.

[CIT0015] Firmansyah, I., Rasni, H. & Rondhianto, 2014, *The relationship between knowledge and preparedness behavior in dealing with flood and landslides in adolescents 15–18 years old at Al-Hasan Kemiri High School*, Panti District, Jember Regency, viewed 01 September 2021, from https://repository.unej.ac.id/bitstream/handle/123456789/60652/Iman%20Firmansyah.pdf?sequence=1.

[CIT0016] Gill, J.C., Ekbal, H. & Bruce, D.M., 2020, *Workshop report: Multi-hazard risk scenarios for tomorrow’s cities tomorrow’s cities urban risk in transition*, UK Research and Innovation GRCF, London.

[CIT0017] Guddo, 2020, ‘The vulnerability of older adults and natural disaster’, *Disaster Advances* 13(11), 79–83.

[CIT0018] Hildayanto, A., 2020, ‘Knowledge and preparedness of the community against flood disasters’, *Higeia Journal of Public Health Research and Development* 4(4), 77–586.

[CIT0019] Islam, F.M.A., Chakrabarti, R. & Dirani, M., 2014, ‘Knowledge, attitudes and practice of diabetes in rural Bangladesh: The Bangladesh population based diabetes and eye study (BPDES)’, *PLoS One* 9(10), 1–11. 10.1371/journal.pone.0110368 PMC4196995 25313643

[CIT0020] Jannah, I., Daniah & Nur, A., 2021, ‘Analysis of older people preparedness to face flood disaster in Kebalen Village, Jambi’, *STIKES Mitra Ria Husada* X(2), 1–11.

[CIT0021] Kar, N., 2016, ‘Care of older persons during and after disasters: Meeting the challenge’, *Journal of Geriatric Care and Research* 3(1), 7–12.

[CIT0022] Kawasaki, H., Satoko, Y., Mio, Y. & Yoshihiro, M., 2022, ‘Introductory disaster training for aspiring teachers: A pilot study’, *Sustainability* 14(6), 1–12.

[CIT0023] Kontan, 2021, *Peta bencana*, viewed 10 June 2022, from https://insight.kontan.co.id/news/peta-bencana.

[CIT0024] Kyoo-Man, H., 2020, ‘Disaster management: From a one sided approach to an inclusive system’, *Environment Systems and Decisions* 40(1), 403–412.

[CIT0025] Lenawida, 2011, ‘The effect of knowledge, attitudes, and support of family members on household preparedness in facing earthquake disasters in Deyah Raya village, Syiah Kuala District, Banda Aceh City,’ Thesis, North Sumatera University, Medan.

[CIT0026] Lesley, G., 2017, ‘Social determinants of health, disaster vulnerability, severe and morbid obesity in adults: Triple jeopardy?’, *International Journal of Environmental & Public Health* 14(12), 1452. 10.3390/ijerph14121452

[CIT0027] Nastiti, R.P., Rafiah, M.P. & Acim, H.I., 2021, ‘Factors related to community preparedness in dealing with flood disasters in Kebon Pala Village, East Jakarta’, *Poltekita: Jurnal Ilmu Kesehatan* 15(1), 48–56. 10.33860/jik.v15i1.219

[CIT0028] National Disaster Management Agency, 2012, *Community based early warning system guidelines*, National Disaster Management Agency, Jakarta.

[CIT0029] National Disaster Management Agency, 2016, *The Indonesian disaster risk*, National Disaster Management Agency, Jakarta.

[CIT0030] National Disaster Management Agency, 2017, *Disaster preparedness handbook building awareness, alertness, and preparedness in facing disasters*, National Disaster Management Agency, Jakarta.

[CIT0031] National Disaster Management Agency, 2021, *The Indonesian disaster risk index*, National Disaster Management Agency, Jakarta.

[CIT0032] Notoatmojo, S., 2007, *Health behavioral science*, Rineka Cipta, Jakarta.

[CIT0033] Nurhidayati, I. & Bahar, K, 2018, ‘Family support increases the preparedness of the older people in the face of volcanic disasters’, *Jurnal Keperawatan Respati Yogyakarta* 5(1), 302–308.

[CIT0034] Oktarina, Fachrudi, H. & Made, A.B., 2009, ‘The relationship between respondent characteristics, regional conditions and knowledge, attitudes towards HIV/AIDS in Indonesian society’, *Buletin Penelitian Sistem Kesehatan* 12(4), 362–369.

[CIT0035] Puspitasari, P., Lukita, D. & Rismayanti, 2021, ‘Cadre development in an effort to increase cadre readiness to face flood disasters in one of the villages in Baleendah District’, *Bina Sehat Masyarakat* 1(1), 14–18.

[CIT0036] Raikes, J., Timothy, F.S., Christine, J. & Claudia, B., 2019, ‘Pre-disaster planning and preparedness for floods and droughts: A systematic review’, *International Journal of Disaster Risk Reduction* 38, 1–9. 10.1016/j.ijdrr.2019.101207

[CIT0037] Sastroasmoro, S. & Ismael, S., 2014, *Fundamentals of clinical research methodology*, CV Sagung Seto, Jakarta, pp. 149–150.

[CIT0038] Sogand, T., Parisa, M.M., Shahnam, S.M., Mohsen, D. & Rahim, A.S., 2019, ‘The importance of education on disasters and emergencies: A review article’, *Journal of Education and Health Promotion* 8(85), 1–7. 31143802 10.4103/jehp.jehp_262_18 PMC6512217

[CIT0039] Tearne, J.E., Bhushan, G., Lajina, G., Jennifer, L. & Newnham, E.A., 2021, ‘The health and security of women and girls following disaster: A qualitative investigation in post-earthquake Nepal’, *International Journal of Disaster Risk Reduction* 66, 102622.

[CIT0040] Teja, M., 2021, *Research center of the Indonesian house of representatives expertise board, community preparedness for vulnerable groups in dealing with natural disasters in Lombok*, viewed 10 September 2021, from https://berkas.dpr.go.id/sipinter/files/sipinter-773-016-20200708131842.pdf.

[CIT0041] The Indonesian Minister for Public Works and Human Settlements, 2017, *Modul of disaster management flood disaster management training*, Water Resources and Construction Education and Training Center, Bandung.

[CIT0042] The Indonesian Ministry of Agraria and Spatial Planning, 2006, *Depok city towards a disaster-resilient and climate change-resilient city*, Directorate General of Spatial Planning, Jakarta.

[CIT0043] United Nations Office for Disaster Risk Management (UNDRR), 2022, *Disaster management*, viewed 15 March 2022, from https://www.undrr.org/terminology/disaster-management.

[CIT0044] United Nations Office for Outer Space Affairs (UNOOSA), 2022, *Disaster management*, viewed 18 March 2022, from https://www.unoosa.org/oosa/en/ourwork/topics/disaster-management.html.

[CIT0045] World Health Organization, 2002, *Gender and health in disasters*, WHO, Geneva.

[CIT0046] Yodsuban, P. & Khanitta, N., 2021, ‘Community-based flood disaster management for older adults in southern of Thailand: A qualitative study’, *International Journal of Nursing Sciences* 8(4), 409–417. 34631991 10.1016/j.ijnss.2021.08.008 PMC8488803

